# EMS injury cause codes more accurate than emergency department visit ICD-10-CM codes for firearm injury intent in North Carolina

**DOI:** 10.1371/journal.pone.0295348

**Published:** 2024-04-30

**Authors:** Nicole L. Snyder, Amy Ising, Anna E. Waller

**Affiliations:** Carolina Center for Health Informatics in the Department of Emergency Medicine, School of Medicine, University of North Carolina at Chapel Hill, Chapel Hill, NC, United States of America; Seattle University, UNITED STATES

## Abstract

**Background:**

The timeliness, accuracy, and completeness of data for firearm injury surveillance is crucial for public health surveillance efforts and informing injury prevention measures. While emergency department (ED) visit data can provide near real-time information on firearms injuries, there are concerns surrounding the accuracy of intent coding in these data. We examined whether emergency medical service (EMS) data provide more accurate firearm injury intent coding in comparison to ED data.

**Methods:**

We applied a firearm injury definition to EMS encounter data in NC’s statewide syndromic surveillance system (NC DETECT), from January 1, 2021, through December 31, 2022. We manually reviewed each record to determine intent, and the corresponding manual classifications were compared to the injury cause codes entered in the EMS data and to ED visit records where EMS-ED record linkage was possible. We then calculated the sensitivity, specificity, positive and negative predictive values for each intent classification in SAS 9.4 using the manually reviewed intent classifications as the gold standard.

**Results:**

We identified 9557 EMS encounters from January 1, 2021, through December 31, 2022 meeting our firearm injury definition. After removing false positives and duplicates, 8584 records were available for manual injury classification. Overall, our analysis demonstrated that manual and EMS injury cause code classifications were comparable. However, for the 3401 EMS encounters that could be linked to an ED visit record, sensitivity of the ED ICD-10-CM codes was low for assault and intentional self-harm encounters at 18.2% (CI 16.5–19.9%) and 22.2% (CI 16–28.5%), respectively. This demonstrates a marked difference in the reliability of the intent coding in the two data sources.

**Conclusions:**

This study illustrates both the value of examining EMS encounters for firearm injury intent, and the challenges of accurate intent coding in the ED setting. EMS coding has the potential for more accurate intent coding than ED coding within the context of existing hospital-based coding guidance. This may have implications for future firearm injury research, especially for nonfatal firearm injuries.

## Introduction

As firearm injuries continue to be a significant cause of morbidity and mortality in the United States (US), public health researchers continue to seek improvements to the timeliness, accuracy, and completeness of firearm injury surveillance [1]. In North Carolina (NC), firearm injury surveillance relies primarily on the NC Violent Death Reporting System (NC-VDRS), hospital administrative data, and syndromic surveillance emergency department visit data [2]. While syndromic surveillance emergency department (ED) visit data provide near real time information on nonfatal firearm injuries in NC, there are concerns about accuracy when subtyping firearm injuries by intent, which includes assault, unintentional (accidental), intentional self-harm, legal intervention and undetermined intent. For example, while some studies estimate unintentional firearm injury to represent 16% or less of all nonfatal firearm injury, approximately 60% of ED firearm injury visits in NC are identified as unintentional based on the ICD-10-CM external mechanism of injury codes [3, 4]. Several other studies that have documented these coding intent discrepancies and the potential overclassification of nonfatal firearm injury as unintentional relied on urban, Level I Trauma center data from a small number of hospitals [5, 6]. For comparison, we aimed to quantify any coding intent discrepancies using data from a statewide system in a predominantly rural state. To achieve this, we compared firearm injury intent classifications identified in NC emergency medical services (EMS) encounter data to NC ED visit data for 2021 and 2022. We manually reviewed all EMS encounters identified as firearm injuries and classified each record by intent. We then compared these intent classifications to EMS injury cause codes, and to the firearm injury intent classifications identified in syndromic surveillance ED visit data for those patients transported to the ED by EMS. Next, we calculated sensitivity, specificity, positive predictive value (PPV), and negative predictive value (NPV) for the manually reviewed dataset to the injury cause codes and for the manually reviewed dataset to the ED visit data. We specifically chose to examine EMS data because precedence [7–11] has shown that EMS narratives can provide rich contextual details on the entire EMS encounter; therefore, the EMS data should provide insights into firearm injury intent.

## Methods

### Study design

This was a statewide, retrospective observational study that investigated the accuracy of nonfatal firearm injury intent in EMS encounter data and ED visit data in NC. We manually reviewed and assigned intent to firearm injuries identified in the EMS encounter data and compared these intents to those coded by EMS providers in the EMS injury cause field. We also linked EMS encounters to ED visit data and compared our manually assigned intents identified in the EMS data to intent based on ICD-10-CM final diagnosis codes assigned in the ED visit data. This study was reviewed by the Office of Human Research Ethics at the University of North Carolina at Chapel Hill and was determined to be exempt from further review according to the secondary data regulatory category under 45 CFR 46.104. As such, consent for this study was waived.

### Study data sources and variables

With approval from the NC Office of Emergency Medical Services, we downloaded all EMS encounters from January 1, 2021 through December 31, 2022 identified as firearm-related injuries from NC’s statewide syndromic surveillance system NC DETECT [12] on February 1, 2023 with an update to incorporate additional records on May 1, 2023. We identified firearm injuries in the EMS data using a rules-based definition that searches the complaint for gunshot wound related injuries OR the injury cause field for firearm-related ICD-10-CM codes. The definition also searched for gunshot wound related injuries in the EMS narrative if the dispatch complaint was *Stab/Gunshot Wound/Penetrating Trauma*. This initial definition was designed to maximize sensitivity, while excluding EMS encounters that are not for active responses based on EMS patient disposition (Table 1).

**Table 1:** EMS firearm V1 definition.

Complaint Terms	GSW or gunshot or "gun shot" or "shot myself" or "shot self*" or firearm or "I got shot" or "been shot" or "shot gun" or shotgun
Injury cause codes	W34, W34.0, W34.09, W34.00, W34.00XA, W33.02, W33.03, W33.09, W33.01, W32.0, W32, W34.1, W33.0, W33, X94.0XXA, X95.9, X95.9XXA, Y22, Y22.XXXA, Y23.1, X72.XXXA, X74.9, Y35.0, Y35.02, Y35.023, Y35.03, Y35.092, Y35.093, Y35.003, Y23.3, Y22.0, Y24.8, Y23.8, Y23, Y23.0, Y23.0XXA, Y38.4X, Y24.9, Y24.9XXA, Y23.9
Dispatch complaint AND narrative	dispatch complaint is Stab/Gunshot Wound/Penetrating Trauma AND narrative keywords of GSW or gun shot or gunshot
Patient disposition exclusions	Assist, Agency; Assist, Public, Assist; Unit, Canceled (Prior to Arrival At Scene); Canceled on Scene (No Patient Contact); Canceled on Scene (No Patient Found); Standby-No Services or Support Provided; Standby-Public Safety, Fire, or EMS Operational Support Provided; Transport Non-Patient, Organs, etc.

The EMS encounters from January 1, 2021 through December 31, 2022 meeting the EMS firearm V1 definition were linked, when possible, to the corresponding ED visit record and provided to the authors for analysis on October 19, 2023. The linkage used an existing hierarchical deterministic process to match records based on drop-off time by EMS, arrival time in the ED, EMS destination name, EMS destination type, patient date of birth, sex and race/ethnicity, ED facility name, and ED transport mode [13]. The linkage rules use the same variables across multiple steps with different matching criteria to maximize the number of linkages while minimizing false linkages. Several updates were made to the linkage process as part of this analysis to increase the likelihood of a successful linkage; we updated the EMS encounter destination type required for linkage to include missing and *Freestanding Emergency Department* destination types as well as *Hospital-Emergency Department*. We also allowed records in the linkage process with missing race and updated the free text list of hospital names used by EMS agencies in the Destination Name field to include additional aliases and misspellings, increasing that list from 3213 to 5739 entries. The authors did not have access to any information that could identify individual participants during or after data collection.

### Data analysis

We manually reviewed each EMS encounter and documented the intent (assault, intentional self-harm, unintentional, undetermined, or legal intervention) in a Microsoft Excel spreadsheet. We also identified false positives. Firearm injury intent was assigned using complaint, narrative, and injury cause code fields. When these fields provided conflicting information, the reviewer used group consensus among the three authors to determine intent. Additional decision points are listed below:

- If injury cause was coded as undetermined, a more precise intent may have been assigned based on the narrative description.
- When the narrative described an incident as a drive-by shooting or shot by someone else while in a car with no documented/undetermined injury cause, intent was classified as assault. This approach is consistent with federal coding recommendations [14–17] and prior studies with manual firearm injury intent classifications.
- If a patient was transported to an ED by EMS, stabilized, and then transferred by EMS to another ED, both EMS encounters were coded. We kept these encounters as part of the initial injury event to maximize comparisons within the EMS data and those transported to an ED, while EMS encounters in our dataset that occurred after surgery or days after the initial injury event were documented as false positive subsequent encounters.
- For deaths at the scene with inconclusive intent in the EMS record, death certificate data were consulted to determine intent, if a linkage could be identified based on patient age, sex, race, county of residence and date of death.

We excluded any encounters identified by the firearm V1 definition that described injuries caused by bb guns, pellet guns, nail guns, air guns, or firearms used as a blunt weapon, e.g., “pistol whip.” We also excluded subsequent encounters for previous firearm injury and near misses.

We compared the EMS manually assigned intent to the intent assigned by EMS providers in the injury cause field based on external mechanism of injury ICD-10-CM codes as well as to the firearm injury intent identified by ICD-10-CM codes assigned in the ED visit data using definitions published by the Council of State and Territorial Epidemiologists (CSTE) (Table 2) [18].

**Table 2:** ICD-10-CM codes for EMS and ED firearm injury intent classifications*.

Intent	ICD-10-CM Codes
Assault	X93, X94, X95.8, X95.9
Intentional Self-Harm	X72, X73, X74.8, X74.9
Legal Intervention	Y35.00-Y35.03, Y35.09
Undetermined	Y22, Y23, Y24.8, Y24.9
Unintentional	W32.0, W32.1, W33.0, W33.1, W34.00, W34.09, W34.10, W34.19

*7^th^ character of A or missing

For each comparison we calculated sensitivity, specificify, PPV and NPV in SAS 9.4. If an EMS encounter received more than one firearm injury cause code, e.g., the same EMS encounter received codes for both assault firearm discharge and unintentional firearm discharge, we counted that record as a true positive if one of the injury cause intents matched the manually assigned intent. If an EMS encounter had more than one injury cause code assigned for firearm injury and none of the codes matched the manually assigned intent, we did not count that record as a true positive for any of the manually assigned intents. Similarly, in the ED visit data, ED visit records that received multiple ICD-10-CM codes with confliciting firearm injury intents were counted as true positives if one of the ICD-10-CM codes matched the manually assigned intent. ED visits were not counted as a true positive for any of the manually assigned intents if none of the confilcting intents in the ED visit data matched a manually assigned intent.

## Results

**Fig 1:**
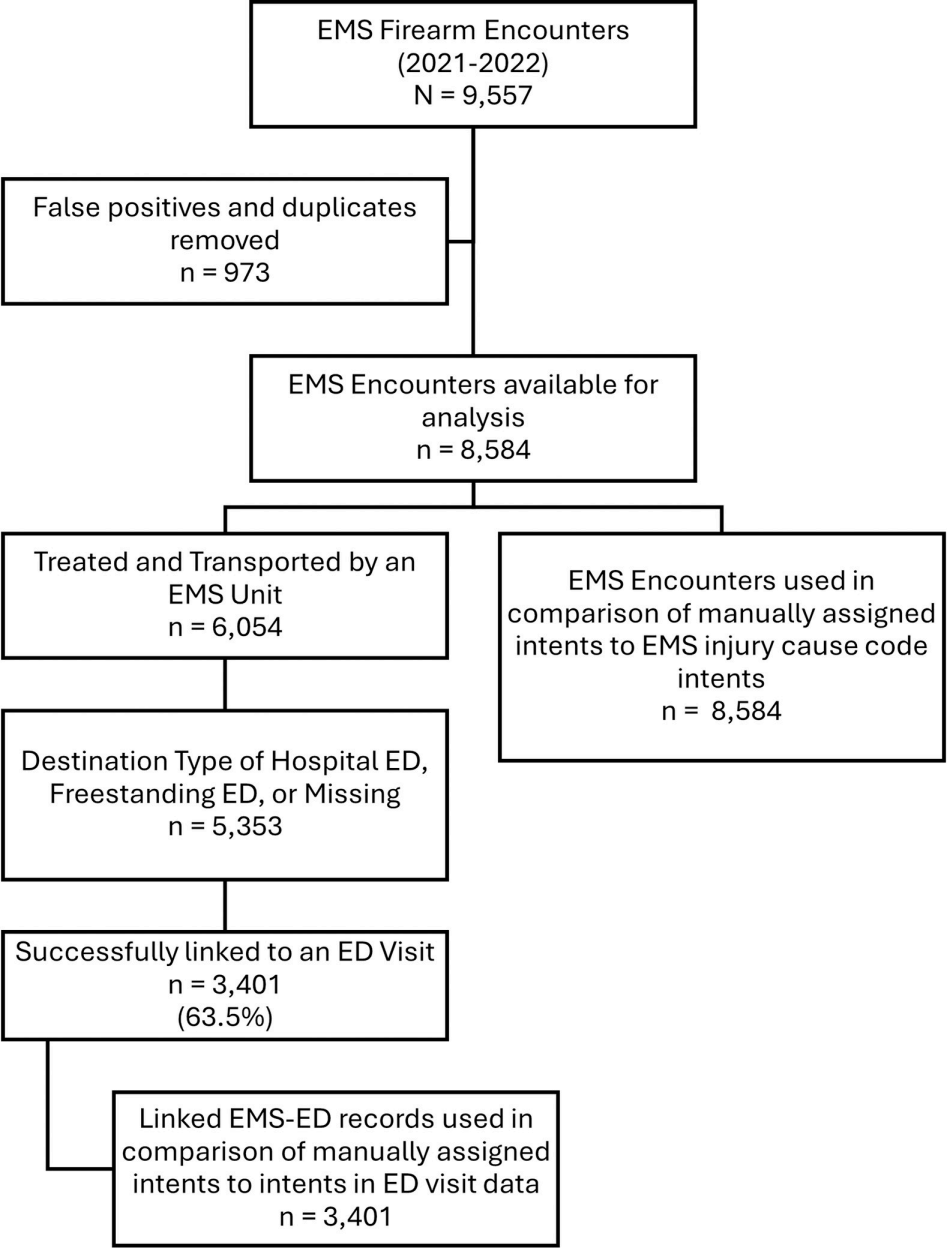
Diagram of data sources used in firearm injury intent comparison.

We manually reviewed 9557 EMS encounters from January 1, 2021, through December 31, 2022 meeting our firearm injury definition. Of these, we identified 969 false positives and four duplicates in our manual review, leaving 8584 EMS encounters for analysis. Of the 8854 EMS encounters included in our analysis, 6054 had a disposition of ‘*Patient Treated*, *Transported by this EMS Unit’* and, of those, 5558 had a destination type of ‘*Hospital-Emergency Department’*, ‘*Freestanding Emergency Department’* or ‘*Missing*’, and 5353 of those were transported to an ED in NC DETECT (i.e., all civilian, acute care EDs in NC), based on a review of the free text destination name. As shown in Fig 1, there were 3,401 (65.5%) eligible EMS encounters successfully linked to and compared by firearm injury intent to corresponding ED visits.

Table 3 summarizes the number of records identified in each dataset grouped by firearm injury intent. Assaults represent the majority of firearm injuries in the manually reviewed EMS data, followed by undetermined, and intentional self-harm encounters. Assaults are also highest when analyzing the EMS data by injury cause code, but there are 2,691 (31.3%) EMS encounters for firearm injuries with either no firearm intent injury cause code or that were missing injury cause codes entirely. In the linked EMS-ED data the intent with the highest number of records is unintentional (58.4%), followed by assault (13.6%). In the linked encounters, 868 (25.5%) ED visits had no ICD-10-CM firearm-related external mechanism of injury code.

**Table 3:** Firearm injury intent by data source, count (%).

Intent	Manually Reviewed EMS Encounters (N = 8584) (Gold Standard) N (%)	EMS Encounters with Firearm Intent Injury Cause Code (N = 8584) N (%)	Firearm Injury Intent in ED visit data for ED visits linked to EMS Firearm Encounters (N = 3401) N (%)
**Assault**	4400 (51.3%)	2953 (34%)	462 (13.6%)
**Intentional Self-Harm**	1581 (18.4%)	1116 (13%)	49 (1.4%)
**Undetermined**	1694 (19.7%)	1218 (14.2%)	29 (0.9%)
**Unintentional**	862 (10%)	599 (7%)	1987 (58.4%)
**Legal Intervention**	47 (0.6%)	7 (.08%)	6 (0.2%)
**Missing Intent / No intent identified**	0 (0%)	2691 (31.3%)	868 (25.5%)

### Manual intent and injury cause code comparison

We compared our manually assigned intent classifications to the EMS injury cause codes (ICD-10-CM) and the results are shown in Table 4.

**Table 4:** Sensitivity, specificity, PPV and NPV of EMS injury cause codes compared to the gold standard (manually assigned intent) percentages and 95% confidence intervals.

Intent	Sensitivity	Specificity	PPV	NPV
Assault	69.4 (68, 70.7)	98.8 (98.4, 99.1)	98.3 (97.9, 98.8)	75.4 (74.3 76.6)
Intentional Self-Harm	67.9 (65.6, 70.2)	99.4 (99.2, 99.6)	96.2 (95.1, 97.4)	93.2 (92.6, 93.8)
Unintentional	64.0 (60.8, 67.2)	99.4 (99.2, 99.6)	92.2 (90.0, 94.3)	96.1 (95.7, 96.5)
Undetermined	33.8 (31.6, 36.1)	90.6 (90.0, 91.3)	47.0 (44.2, 49.9)	84.8 (84.0, 85.6)
Legal Intervention	6.4 (0, 13.4)	100 (99.9, 100)	42.9 (6.2, 79.5)	99.5 (99.3, 99.6)

The EMS encounter data has modest sensitivity in identifying assault, intentional self-harm, and unintentional events, and low sensitivity in identifying undetermined and legal intervention firearm injuries in comparison to the manually assigned intents in the EMS data. However, the specificity is high across all intents with a modest reduction in specificity for undetermined cases.

#### Manual intent and ED visit data comparison

Results of the comparison of ED firearm injury intent to the manually assigned intent in the EMS data are shown in Table 5.

**Table 5:** Sensitivity, specificity, PPV and NPV of ED visit firearm injury intent compared to the gold standard (manually assigned intent) Percentages and confidence intervals.

Intent	Sensitivity	Specificity	PPV	NPV
Assault	18.2 (16.5,19.9)	92.5 (91.1,93.8)	76.0 (72.1–79.9)	46.2 (44.4–48.0)
Intentional Self-Harm	22.2 (16.0, 28.5)	99.7 (99.5, 99.9)	77.6 (65.9,89.2)	96.0 (95.4, 96.7)
Unintentional	73.4 (69.6, 77.3)	44.2 (42.4, 46.0)	18.8 (17.1, 20.5)	90.5 (88.9, 92.0)
Undetermined	1.4 (0.6, 2.3)	99.3 (99.0, 99.6)	37.9 (20.3, 55.6)	77.3 (75.9, 78.7)
Legal Intervention	13.3 (0, 30.5)	99.9 (99.8, 100)	33.3 (0, 71.1)	99.6 (99.4, 99.8)

The ED visit data have very low sensitivity in identifying assault, intentional self-harm, undetermined and legal intervention firearm injuries in comparison to the manually assigned intents in the EMS data. When assault, intentional self-harm, undetermined and legal intervention firearm injury ICD-10-CM codes are used in the ED, however, they have high specificity (92.5%-99.9%).

## Discussion

In this study, EMS data were significantly better at identifying firearm injury intent compared to ED visit data and should be prioritized for timely public health surveillance of firearm injuries. Firearm injury surveillance in NC has recently expanded to include EMS encounter data as they provide more accurate estimates of firearm injuries grouped by assault, unintentional, and intentional self-harm. The ICD-10-CM Official Guidelines for Coding and Reporting state that injury intent coding for ED visits should default to unintentional (accidental) when the intent is not clearly documented by the physician in the medical record data [19]. Based on our findings, this guidance is resulting in an overestimation of unintentional firearm injury and an underestimation of assault and intentional self-harm events in ED visit data.

To address the inaccurate hospital coding for firearm injuries, there are proposals currently under review by the ICD committee to change the default intent for firearm injuries from unintentional (accidental) to assault (Option 1) or to undetermined (Option 2) [20]. While defaulting to undetermined presents challenges when conducting surveillance for firearm injury by intent, defaulting to assault has the potential to significantly overestimate the number of assault firearm injuries, which may result in confusing firearm injury trends disseminated by public health agencies and researchers. Prior research estimated that assaults represent 68.2% of firearm injuries in the hospital setting, but this study relied on data from three urban Level 1 US trauma centers [5]. By comparison, our study leveraged statewide data and classified 25% fewer assaults, or 51.3%, reflecting a significant difference in the types of firearm injuries that can occur across a statewide population. Overestimating assault firearm injury could have serious unintended consequences for victims, public health funding, and appropriate resource allocation for injury prevention programs. Recognizing that the number of unintentional firearm injuries is currently overestimated in the ED setting, a proposal to replace one type of misclassification with another does not solve the ICD-10-CM coding problem regarding firearm injury intent.

In addition, there is concern that defaulting to assault may negatively impact the language used in discussions around firearms injuries, resulting in further stigmatization of racial and/or ethnic communities that are already disproportionately impacted by firearm-related violence. Several recent studies [21–23] have revealed that the higher firearm injury rates frequently associated with many of these communities result in victim blaming without recognizing the underlying trauma and structural racism experienced within them. This, in turn, leads to biased perceptions of firearm-related violence and further stigmatization. Accurate identification of firearm injury intent is a key mechanism for properly understanding the nature of gun violence and characterizing associated challenges.

This study has implications for stakeholders in NC who seek a more comprehensive understanding of firearms injury at the state and local level to develop more targeted interventions. Our results also suggest that states like NC with more rural populations might obtain a more accurate overview of firearms injuries by incorporating EMS surveillance as part of a more comprehensive injury surveillance program. In NC, a legislated safe firearms storage campaign is underway statewide. Information from this study will be used to help guide the messaging for those efforts, but limited funding for firearm injury prevention constrains the reach and impact of these programs.

While this study did not include a comprehensive review of EMS encounters transported to an ED that were not successfully linked to an ED visit record, our review of missed linkages revealed that missing or inaccurate dates of birth represented a significant cause of missed linkages. For serious events such as firearm injury, a reliable date of birth may be challenging for EMS to record at the scene.

## Limitations

Our study has several limitations. When manually coding intent, we relied on the EMS narrative but if the reporting of the incident intent to or by EMS was inaccurate, then the resulting intent coding may be inaccurate. For example, if the patient stated that they were shot in a drive-by shooting we coded this EMS encounter as an assault, but the patient may have unintentionally shot themselves and reported it as a drive-by shooting. In addition, our descriptive analysis included both nonfatal and fatal EMS encounters. While this reflects the comprehensiveness of EMS data for firearm injury, certain findings may be different when analyzing nonfatal and fatal EMS firearm injury encounters separately. Finally, most of the EMS encounters in this analysis were reviewed by only one reviewer; intent classifications may have been assigned differently by a different reviewer.

While our findings demonstrate the value of using EMS injury cause codes for surveillance of firearm injuries by intent, this study was limited by the significant missingness of injury cause codes in our EMS dataset (31.3%). EMS providers should be strongly encouraged to document injury cause codes for all injury-related encounters. Additionally, while our EMS to ED linkage rate was 63.5%, challenges exist in cases of severe injury encounters where the patient may be unconscious and reliable demographic information needed for linkage cannot be obtained. We assert that our findings have merit in identifying the inaccuracies of ED intent coding, while also illustrating that assault-based firearm injuries in a statewide EMS dataset are not as high as those seen in urban trauma centers [5]. Finally, while NC EMS agencies and hospitals follow national standards and guidelines when documenting firearm injuries, our results may not be generalizable outside of NC.

## Conclusions

Timely and accurate injury cause codes are crucial for understanding the nature and scope of firearm-related injuries. In this study, we manually assigned intent to 8584 eligible NC EMS encounters from 2021 and 2022 to better understand the landscape of EMS-related firearm injury calls and to assess the accuracy of firearm injury coding in the ED setting. Manual coding of firearm injury intent revealed comparable results to those obtained from examining EMS assigned injury cause code classifications, but the high degree of injury cause code missingness needs to be addressed to improve sensitivity. Based on our findings, the firearm injury intent coding in the ED data is unreliable and reflects federal coding guidance to default injury intent to unintentional if the intent is not identified in the physician documentation [19].

The outcomes of our study reveal that EMS encounter data may be useful for obtaining more granular data from the scene of the injury incident. Such data might be important for developing tailored approaches that reflect regional and demographic differences in firearms injuries. In contrast, the lack of such detailed information in the ED visit data may limit such interventions. For example, if an encounter is the result of an assault, but it is coded as unintentional or undetermined, a patient may not be flagged for post-discharge follow-up that is directed towards supporting victims of assault. Until firearm injury intent coding in the ED setting is improved, EMS data provide a more reliable source of timely information on firearm injury intent.
